# Cultivable Fungi in Amazonian Black-, White-, and Clear-Water Rivers

**DOI:** 10.3390/biology15120931

**Published:** 2026-06-15

**Authors:** Vanessa da Silva Bindá, Juan Diego Ribeiro de Almeida, Adriana dos Santos Arévalo, Marcelo Bentes de Siqueira, Roberto Moreira da Silva, Ana Claudia Alves Cortez, Eveleise Samira Martins Canto, Érica Simplício de Souza, Hagen Frickmann, João Vicente Braga de Souza

**Affiliations:** 1 Postgraduate Program in Biotechnology and Natural Resources of the Amazon, Amazonas State University (UEA), Manaus 69050-020, Brazil; vanessadasilvabinda@gmail.com (V.d.S.B.); adsa.mbt25@uea.edu.br (A.d.S.A.); esdsouza@uea.edu.br (É.S.d.S.); 2 Mycology Laboratory, National Institute of Amazonian Research (INPA), Manaus 69060-001, Brazil; jdra.dbm23@uea.edu.br (J.D.R.d.A.); marjunior2000@outlook.com (M.B.d.S.J.); robertomsjr@gmail.com (R.M.d.S.J.); accortez@inpa.gov.br (A.C.A.C.); 3 Institute of Biodiversity and Forests, Federal University of Western Pará (UFOPA), Santarém 68035-110, Brazil; eveleise.canto@ufopa.edu.br; 4 Department of Microbiology and Hospital Hygiene, Bundeswehr Hospital Hamburg, 20359 Hamburg, Germany; 5 Institute of Medical Microbiology, Virology and Hygiene, Rostock University Medical Center, 18057 Rostock, Germany

**Keywords:** Amazon basin, aquatic fungi, cultivable diversity, freshwater ecology, hydrochemical gradients, submerged wood, sediment mycobiota

## Abstract

Cultivable fungi are important components of freshwater ecosystems, yet their diversity in Amazonian aquatic environments remains poorly explored. This study evaluated cultivable fungal communities associated with water, sediment, and submerged wood in the Negro, Solimões, and Tapajós Rivers, representing the major black-, white-, and clear-water systems of the Amazon basin, respectively. Samples were collected along 25 m transects, fungi were isolated on potato dextrose agar, grouped into morphotypes, and identified morphologically. Diversity was assessed using richness, Shannon, Simpson, Pielou, and Sørensen indices. Overall, 130 isolates and 75 morphotypes were recorded, with a predominance of morphotypes assigned to filamentous Ascomycota and widespread occurrence of *Aspergillus* and *Penicillium*. Solid substrates yielded greater fungal abundance and richness than water samples. Submerged wood from the Negro River showed the highest overall cultivable fungal abundance, whereas the Tapajós River showed the highest diversity in water and sediment and the highest richness in both substrates. The Solimões River showed stronger dominance by a limited number of morphotypes, particularly in submerged wood. Sørensen similarity values indicated low compositional overlap among rivers, especially for submerged wood communities, suggesting apparent differentiation among river-associated cultivable fungal assemblages. Together, these exploratory results suggest that substrate type, hydrochemical differences, and potential temporal effects may be associated with the structure of cultivable fungal communities in Amazonian rivers.

## Simple Summary

Amazonian rivers differ markedly regarding the chemical composition of their water, but their cultivable fungal communities remain poorly documented, especially when multiple substrates are compared within the same study. Here, cultivable fungi were isolated from water, sediment, and submerged wood collected in three major Amazonian rivers representing black, white, and clear waters: the Negro, Solimões, and Tapajós. Solid substrates, particularly submerged wood and sediment, consistently supported higher fungal abundance and richness than water. A total of 130 isolates and 75 morphotypes were recovered, with a predominance of filamentous Ascomycota and recurrent occurrence of *Aspergillus* and *Penicillium* morphotypes. Diversity patterns varied according to substrate and river system, although these patterns should be interpreted considering the non-synchronized sampling months: submerged wood from the Negro River showed the highest overall fungal abundance and high diversity, the Solimões River showed stronger dominance by a limited number of morphotypes, and the Tapajós River showed the highest diversity in water and sediment. Low similarity among rivers, especially in submerged wood, suggests apparent differentiation among cultivable fungal morphotype assemblages. These exploratory results indicate that substrate type and hydrochemical gradients may contribute to cultivable fungal diversity patterns in Amazonian freshwater ecosystems.

## 1. Introduction

The Amazon basin contains the largest river network on earth and supports one of the highest levels of biodiversity ever documented. Within this ecosystem, aquatic microbial communities play essential roles in biogeochemical cycling, and fungi contribute directly to organic matter decomposition, nutrient recycling, and energy flow in freshwater ecosystems [1,2,3].

Beyond their ecological relevance, cultivable fungi from aquatic environments represent valuable sources of enzymes, metabolites, and other biotechnologically relevant compounds. Understanding how environmental heterogeneity shapes these communities is therefore important not only for freshwater ecology but also for bioprospecting strategies in tropical ecosystems [4,5].

Despite their importance, fungal diversity in Amazonian aquatic habitats remains underestimated. This gap is particularly notable because the region is defined by pronounced hydrochemical contrasts, classically represented by black-, white-, and clear-water rivers, which act as environmental filters for microbial communities [6,7,8]. At a broader scale, current estimates suggest that the number of described fungal taxa still represents only a fraction of the total global fungal diversity [9].

Studies from other freshwater regions have shown that cultivable fungi colonize water, sediment, submerged wood, and plant debris, often exhibiting high taxonomic diversity and frequent dominance of Ascomycota and Basidiomycota. Community structure is influenced by abiotic factors such as temperature, nutrient availability, and hydrological conditions [10,11,12,13]. In addition, fungi recovered from surface and irrigation waters may include opportunistic or potentially pathogenic taxa, reinforcing the ecological and sanitary relevance of freshwater mycobiota surveys [14,15].

In the Amazon, however, most available studies have focused on submerged wood, Ingoldian fungi, or isolates screened for bioactive compounds, whereas few investigations have simultaneously compared water, sediment, and submerged wood across river systems with contrasting water types [16,17,18,19,20]. Consequently, the interactive effects of substrate heterogeneity and river hydrochemistry on cultivable fungal communities remain poorly understood at a regional scale.

Here, we investigated cultivable fungi associated with water, sediment, and submerged wood in the Negro, Solimões, and Tapajós Rivers. We hypothesized that (i) solid substrates would harbor greater fungal richness and abundance than water samples and (ii) differences among black-, white-, and clear-water systems would be reflected in fungal diversity patterns and community composition.

## 2. Materials and Methods

### 2.1. Study Design

This study consisted of a laboratory-based, descriptive-comparative investigation with a quantitative approach aimed at evaluating the diversity of cultivable fungi recovered from environmental samples collected from different compartments of Amazonian fluvial systems, namely water, sediment, and submerged wood. The analytical approach included the isolation of cultivable fungal propagules, morphological characterization of the recovered isolates, quantification of colony-forming units, and ecological assessment of the community structure using descriptive diversity metrics. All laboratory procedures were carried out at the Mycology Laboratory of the National Institute of Amazonian Research (INPA), Manaus, Amazonas, Brazil.

### 2.2. Study Area

**Figure 1:**
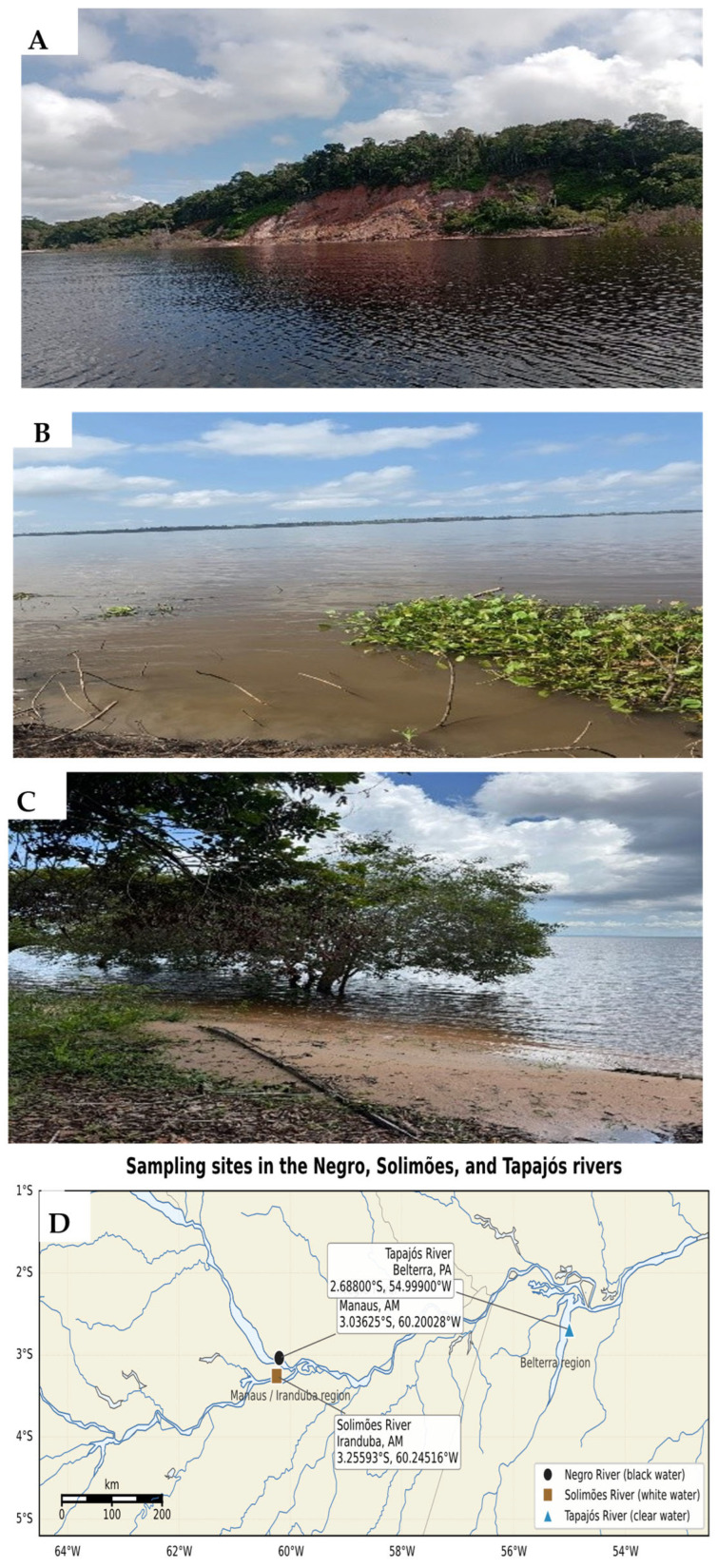
Geographic location of the sampling sites in the Negro, Solimões, and Tapajós rivers, Brazilian Amazon. (**A**) Sampling site in the Negro River near Manaus, Amazonas, Brazil (−3.03625, −60.20028). (**B**) Sampling site in the Solimões River near Iranduba, Amazonas, Brazil (−3.255930, −60.245157). (**C**) Sampling site in the Tapajós River near Belterra, Pará, Brazil (−2.68800, −54.99900). (**D**) Map showing the location of the three sampling sites in the Brazilian Amazon. Base maps were obtained from the Google Earth web version (Google LLC, Mountain View, CA, USA; accessed in 2025). Blue lines represent the main river channels displayed in the base map. The figure was as-sembled by the authors.

Sampling was conducted in three major Amazonian rivers with contrasting physicochemical characteristics: the Solimões River, in the municipality of Iranduba, Amazonas (AM), Brazil (−3.255930, −60.245157); the Negro River, near Manaus, Amazonas (AM), Brazil (−3.03625, −60.20028); and the Tapajós River, in the region of Belterra, Pará (PA), Brazil (−2.688, −54.999). These fluvial systems were selected because they represent distinct Amazonian water types, namely white-water, black-water, and clear-water environments, respectively, thereby allowing a comparative assessment of fungal diversity across contrasting ecological settings. The above-mentioned geographic coordinates correspond to the central point of each sampling area (Figure 1).

Collections from the Negro River were performed in April, those from the Tapajós River in May, and those from the Solimões River in September 2025. Samples from the Tapajós River were collected by Eveleise Samira Martins Canto and collaborators, maintained at 5 °C during transport, and subjected to cultural growth in Manaus within 24 h after collection. In contrast, samples from the Negro and Solimões Rivers were processed within less than 2 h after collection and maintained at room temperature until processing. This study consisted of an exploratory cross-sectional comparison among hydrochemically contrasting river systems; therefore, seasonal replication was beyond the scope of the present investigation. Access to Brazilian genetic heritage associated with the collection of fungal samples was registered in the National System for the Management of Genetic Heritage and Associated Traditional Knowledge (SisGen), under registration No. A9A738C.

### 2.3. Sampling Design and Collection of Environmental Samples

For each river, a geographic reference point was established, and a 25 m stretch along the riverbank was delimited. Within each transect, five subsamples were collected for each substrate type, namely water, sediment, and submerged wood, with an approximate distance of 5 m between adjacent sampling points. This design resulted in 15 samples per river and 45 samples in the overall study.

Water samples were collected at approximately 0.5 m depth to obtain material representative of the surface water column. Sediment samples were collected using a sterile 50 mL polypropylene conical tube (Falcon^®^, Corning Inc., Corning, NY, USA), from which approximately 50 g of material was obtained at a depth of about 1 m. For submerged wood, fragments of variable size were selected, prioritizing submerged or partially submerged material showing visible signs of active decomposition. All samples were handled carefully to minimize cross-contamination and placed in sterile containers. Samples from the Negro and Solimões Rivers were kept at approximately 26 °C and processed within less than 2 h after collection. Samples from the Tapajós River were transported under refrigerated conditions at 5 °C and processed approximately 24 h after arrival in Manaus. Subsequently, all materials were subjected to laboratory analyses.

### 2.4. In Situ Physicochemical Characterization of Water

The physicochemical parameters of the water were measured in situ using a previously calibrated multiparameter probe ProfLine 197i (WTW/Xylem Analytics Germany Sales GmbH & Co. KG, Weilheim, Germany) according to the manufacturer’s recommendations. The parameters measured included temperature (°C), pH, electrical conductivity (µS/cm), and dissolved oxygen (mg/L). These variables were recorded at the time of sample collection in order to characterize the environmental conditions associated with each fluvial system and to support the ecological interpretation of the cultivable fungal communities recovered from the different rivers. For the Solimões River, dissolved oxygen and electrical conductivity data were complemented with values reported in the literature to strengthen the hydrochemical characterization of the sampled site [21,22].

Water samples were additionally analyzed by UV–Vis spectrophotometry to assess turbidity and absorbance profiles. Turbidity was estimated by absorbance at 600 nm (A600), and spectral scans were obtained between 200 and 700 nm using a spectrophotometer (Eppendorf AG, Hamburg, Germany) equipped with quartz cuvettes of 10 mm. Sterile distilled water was used as the blank. These measurements complemented the physicochemical characterization of the sampled river systems.

### 2.5. Isolation of Cultivable Fungi

#### 2.5.1. Culture Medium and General Isolation Strategy

Cultivable fungi from water, sediment, and submerged wood were isolated by serial dilution followed by surface spreading on potato dextrose agar (PDA) medium (Difco, Becton, Dickinson and Company, Franklin Lakes, NJ, USA) supplemented with chloramphenicol (Sigma-Aldrich, St. Louis, MO, USA) at a final concentration of 200 mg/L. Chloramphenicol was added to reduce bacterial growth and facilitate fungal recovery from environmental samples with high microbial complexity.

#### 2.5.2. Sample Processing and Serial Dilution

For water samples, 1 mL of each sample was transferred aseptically to 9 mL of sterile saline solution (0.85% NaCl), resulting in the initial dilution of 10^−1^. For sediment and submerged wood samples, 1 g of material was homogenized in 9 mL of the same sterile saline solution. Subsequent serial dilutions were adjusted according to the physical characteristics of each substrate and the expected microbial load, reaching 10^−2^ for sediment samples and 10^−3^ for wood samples. For plating, two dilutions were selected for each substrate type: 10^0^ and 10^−1^ for water, 10^−1^ and 10^−2^ for sediment, and 10^−2^ and 10^−3^ for submerged wood. This strategy was adopted to improve the recovery of countable colonies while maximizing the detection of morphologically distinct cultivable taxa across the various substrates evaluated.

#### 2.5.3. Plating, Incubation, and Recovery of Isolates

Aliquots of 100 µL from each selected dilution were spread onto Petri dishes containing PDA supplemented with chloramphenicol. Plates were incubated at 25 °C for 7–14 days and monitored daily for colony development. The incubation period was selected to allow the recovery of both fast- and slow-growing cultivable morphotypes. In addition to quantitative assessment, all dilutions streaked out on agar plates were examined for fungal recovery and morphotype categorization, thereby increasing the likelihood of detecting taxa that might be underrepresented at a single dilution level.

After primary growth, colonies showing distinct macromorphological features were sub-cultured repeatedly until pure cultures were obtained. These purified isolates were subsequently used for morphological characterization and for the construction of abundance matrices employed in the ecological analyses. Although the primary quantitative estimates were based on the dilution that yielded the most suitable countable colony range for each sample, all plates were also examined qualitatively to minimize losses in detectable phenotypic diversity.

Because cosmopolitan genera such as *Aspergillus*, *Penicillium*, *Cladosporium*, and *Fusarium* may occur in environmental samples and may also be associated with laboratory contamination, their inclusion in the dataset was based on recovery from processed environmental samples, purification of colonies, and reproducible macro- and micromorphological features. These isolates were interpreted conservatively as cultivable environmental morphotypes recovered under the conditions used in this study.

### 2.6. Estimation of Fungal Abundance

The abundance of cultivable fungi was estimated by counting colony-forming units (CFU), expressed as CFU/mL for water samples and CFU/g for sediment and submerged wood samples. Colony counts obtained from the selected plates were corrected according to the dilution factor and inoculated volume, using the equation: CFU = (number of colonies × dilution factor)/inoculated volume. An inoculated volume of 0.1 mL was considered for all plated samples. For each river and each substrate type, five subsamples were analyzed. Mean abundance values and standard deviations were then calculated to represent local spatial variation along the sampling transect.

For each cultivable fungal unit recognized as a morphotype, abundance was calculated as the arithmetic mean of the five subsamples from the same substrate and river. When a morphotype was detected in only one subsample, zero values from the remaining subsamples were retained for the calculation of mean values to avoid overestimation of mean abundance values. The abundance analyses included morphotypes assigned to identified genera and morphologically unresolved fungal categories, such as sterile mycelia, indeterminate isolates, and yeast-like morphotypes.

### 2.7. Morphological Identification of Cultivable Fungi

#### 2.7.1. Macromorphological Characterization

Macromorphological characterization was based on classical taxonomic criteria widely used in mycology, including colony diameter, growth rate, topography, surface texture, colony margin, pigmentation of the obverse and reverse, exudate production, and the presence or absence of diffusible pigments in the culture medium. These traits are useful for the preliminary delineation of commonly recovered environmental filamentous fungi, particularly genera such as *Aspergillus*, *Penicillium*, *Trichoderma*, *Mucor*, *Rhizopus*, and *Cladosporium*. In the present study, colony-level traits were recorded systematically during incubation and interpreted together with micromorphological observations to improve genus-level identification and the recognition of distinct morphotypes [23,24,25].

#### 2.7.2. Micromorphological Characterization by Slide Culture

Micromorphological analysis was performed using the slide culture technique. Small fragments of fungal colonies were transferred to PDA blocks previously arranged in a moist chamber on microscope slides. The blocks were covered with sterile coverslips and maintained at 25 ± 2 °C for 5 to 7 days, or until reproductive structures became visible. After incubation, the coverslips were carefully removed and mounted on glass slides containing lactophenol cotton blue stain (Sigma-Aldrich, St. Louis, MO, USA), following standard mycological procedures [23]. Microscopic examination was performed using a light microscope (Axioscope; Carl Zeiss Microscopy GmbH, Oberkochen, Germany) with 10×, 20×, 40×, and 100× objectives, depending on the structures being examined. The structures evaluated included hyphae, conidia, conidiophores, sporangia, sporangiospores, septation patterns, and other vegetative or reproductive structures when present. Identification was attempted to the genus level whenever possible by comparison with classical and contemporary taxonomic references, including standard mycology manuals and atlas-based resources for filamentous fungi and yeasts [23,24,25,26]. This approach was adopted because the study focused on the structure of cultivable fungal communities and the ecological distribution of phenotypically distinct taxa rather than on species-level molecular delimitation.

Representative images of agar plates, purified colonies, and micromorphological structures of selected isolates are organized as Supplementary Figures S1–S4. These images include representative morphotypes from the most frequent genera recovered in the study, as well as selected low-frequency morphotypes when diagnostic structures were available. The images were included to document the morphological basis used for morphotype recognition and genus-level assignment. Scale bars were added to microscopic images whenever applicable.

Morphological analyses were performed at the INPA Mycology Laboratory, which has longstanding experience with the isolation, cultivation, and micromorphological characterization of filamentous fungi from environmental and clinical sources. Genus-level assignments were based on the combined evaluation of colony morphology and microscopic reproductive structures, following classical and contemporary taxonomic references. Because the purpose of the study was to compare cultivable fungal assemblages among substrates and river systems, isolates were conservatively treated as morphotypes rather than as species confirmed by molecular methods.

#### 2.7.3. Definition of Morphotypes and Unresolved Categories

Because isolates assigned to the same genus may exhibit considerable phenotypic variation, morphological diversity was established based on the recognition of distinct morphotypes. Isolates attributed to the same genus were classified as different morphotypes when they exhibited consistent and reproducible differences in colony pigmentation, surface appearance, texture, border pattern, growth behavior, or micromorphological structure, even when sharing the same general taxonomic assignment. Cultures that failed to develop diagnostic reproductive structures after successive subculturing were classified as sterile mycelia. Isolates with insufficient structures for reliable identification were classified as indeterminate. Isolates showing predominantly yeast-like growth, but lacking diagnostic features for genus-level identification, were grouped as yeast-like morphotypes.

### 2.8. Ecological Analysis of Fungal Community Diversity

#### 2.8.1. Construction of Abundance Matrices

To evaluate community structure, abundance matrices were constructed using the mean CFU values of each morphotype for each river–substrate combination, based on the corrected abundance values obtained from the plated samples. These abundance vectors were used to calculate richness, Shannon diversity, Simpson dominance, and Pielou evenness, whereas Sørensen similarity was calculated separately from presence–absence data for each substrate type.

#### 2.8.2. Alpha Diversity

Alpha diversity was estimated using the Shannon–Wiener diversity index (H′), Simpson dominance index (D), and Pielou evenness index (J). These indices have been widely applied in studies of freshwater fungal communities to describe richness, abundance distribution, and community evenness under contrasting environmental conditions [16,27,28]. The Shannon–Wiener index was calculated as H′ = −Σ(pi ln pi), where pi corresponds to the proportion of individuals belonging to morphotype i relative to the total number of individuals in the sample. This index was used to express diversity by simultaneously considering richness and the distribution of abundance. Simpson dominance was calculated as D = Σpi2 and was used to evaluate the concentration of abundance within a limited number of morphotypes, with higher values indicating stronger dominance. Pielou evenness was calculated as J = H′/ln(S), where S is the total number of morphotypes recorded in the analyzed matrix. This index was used to estimate how uniformly abundances were distributed among morphotypes within each community.

#### 2.8.3. Similarity Among Communities

Community compositional similarity was evaluated using the Sørensen coefficient (Cs), from presence–absence data using Cs = 2a/(2a + b + c), where “a” corresponds to the number of morphotypes shared between two communities, “b” to the number of morphotypes exclusive to the first community, and “c” to the number of morphotypes exclusive to the second community. For each substrate type, similarity values were calculated for the river pairs Negro versus Solimões, Negro versus Tapajós, and Solimões versus Tapajós, considering the total set of morphotypes recorded for each fluvial system. Higher coefficient values indicated greater sharing of morphotypes between the assessed communities.

### 2.9. Data Analysis

All analyses were conducted descriptively in the R statistical environment (version 4.5.1; R Foundation for Statistical Computing, Vienna, Austria), using the vegan package (version 2.7-5). The abundance matrices generated from the morphotype counts served as the basis for calculating richness, diversity, dominance, evenness, and similarity indices. No inferential statistical analyses were performed because the primary objective of the study was to describe patterns of community structure and composition across rivers and substrates rather than to test formal differences among groups.

## 3. Results

### 3.1. Physicochemical Characterization of the River Systems

The three river systems showed physicochemical differences consistent with the classical distinction between black-, white-, and clear-water rivers. Water temperature ranged from 29.8 ± 1.5 °C in the Negro River to 31.7 ± 1.6 °C in the Tapajós River. The Negro River was more acidic (pH 5.53 ± 0.28), whereas the Solimões and Tapajós Rivers showed values closer to neutrality (6.65 ± 0.33 and 6.59 ± 0.33, respectively).

Electrical conductivity was highest in the Solimões River (~80–100 µS·cm^−1^), in contrast to the Negro (9.30 ± 0.47 µS·cm^−1^) and Tapajós (13.72 ± 0.69 µS·cm^−1^) rivers. Turbidity (A600) ranged from 0.015 ± 0.019 to 0.032 ± 0.009, and dissolved oxygen varied from 4.1 to 6.1 mg/L across the sampled sites.

**Figure 2:**
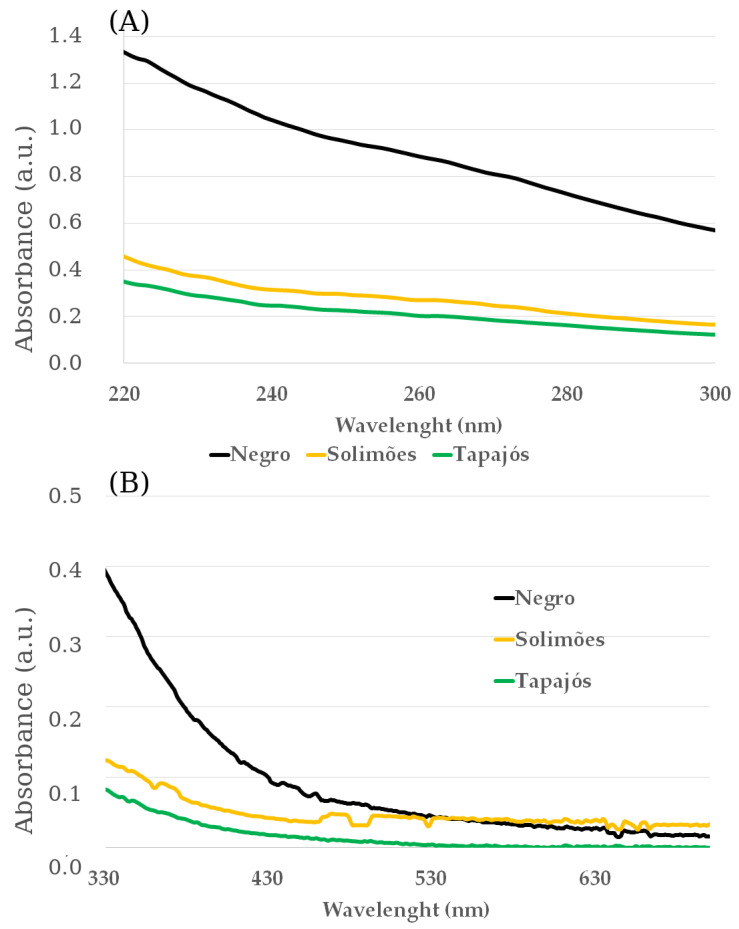
Absorbance profiles of water samples from the three river systems. (**A**) Absorbance profiles in the 200–300 nm range. (**B**) Absorbance profiles in the 330–700 nm range. The Negro River showed the highest absorbance values, particularly in the ultraviolet region.

Spectrophotometric profiles also differed among the assessed rivers. The Negro River showed the highest absorbance in the 200–300 nm range, reaching approximately 2.0 a.u. at 200 nm, and also retained higher absorbance than the other rivers between 330 and 700 nm. These physicochemical characteristics are summarized in Table 1, while the absorbance profiles are presented in Figure 2.

### 3.2. Abundance of Cultivable Fungi Across Substrates and Rivers

Cultivable fungi were recovered from all substrates; however, abundance was consistently higher in solid substrates than in water. These results are presented in Table 2.

The highest total abundance was observed in wood samples from the Negro River (3.4 × 10^4^ ± 0.9 × 10^4^ CFU/g). In sediment, the highest value was recorded in the Solimões River (8.2 × 10^3^ ± 9.5 × 10^3^ CFU/g). In water samples, the Tapajós River showed the highest fungal abundance (140 ± 177 CFU/mL), whereas the Negro River showed the lowest value (22 ± 4.5 CFU/mL).

### 3.3. Taxonomic and Morphological Composition

A total of 130 isolates corresponding to 75 morphotypes were recovered. These isolates were distributed across 16 identified genera and over three additional morphological categories (sterile mycelia, undetermined morphotypes, and yeast-like morphotypes).

Morphotype richness varied among rivers and substrates. The Tapajós River showed the highest richness in water (13 morphotypes) and sediment (22 morphotypes), whereas the Negro River showed the highest richness in submerged wood (22 morphotypes). In all rivers, sediment and wood consistently yielded higher richness than water.

The identified genera included *Aspergillus*, *Penicillium*, *Cladosporium*, *Fusarium*, *Trichoderma*, *Rhizopus*, *Acremonium*, *Exophiala*, *Nodulisporium*, *Scedosporium*, *Scopulariopsis*, *Mucor*, *Oidiodendron*, *Phoma*, *Paecilomyces*, and *Colletotrichum*. Although morphotype richness was high, the number of genus-level identifications and morphological categories was lower, reflecting the occurrence of multiple morphotypes within the same genus.

Representative images supporting the morphological characterization are provided in the Supplementary Figures S1–S4. These figures include examples of agar plates used for primary isolation, purified colonies, and micromorphological structures of selected morphotypes. These images illustrate the phenotypic criteria used to separate isolates into morphotypes and to assign representative isolates on genus level.

Most morphotypes were restricted to one river or substrate, with limited overlap among river systems. A smaller number of morphotypes occurred in more than one river, indicating partial overlap in distribution. Shared morphotypes were more frequent between the Solimões and Tapajós Rivers, whereas the Negro River exhibited a distinct profile.

The distribution of morphotypes across substrates and rivers is summarized in Table 3. Morphotypes also varied quantitatively across substrates and rivers.

### 3.4. Quantitative Structure of the Cultivable Fungal Community

Mean abundance values of morphotypes varied among substrates and river systems (Table 4, Table 5 and Table 6).

High standard deviations were observed for several morphotypes because abundance values were calculated across five subsamples. Many morphotypes occurred in only one or a few subsamples. Zero values from subsamples in which a morphotype was not detected were retained in the calculation. Consequently, repeated values such as 200 ± 447 reflect the same abundance pattern across subsamples, typically detection in a single subsample and absence from the remaining ones.

In submerged wood (Table 4), the highest abundances were recorded for *Acremonium* MT040 in the Solimões River (6.8 × 10^3^ ± 15.2 × 10^3^ CFU/g), *Aspergillus* MT001 in the Negro River (6.4 × 10^3^ ± 9.2 × 10^3^ CFU/g), and *Penicillium* MT028 in the Tapajós River (4.4 × 10^3^ ± 6.2 × 10^3^ CFU/g).

In sediment (Table 5), the highest density values in the Solimões River were assigned to *Penicillium* morphotypes (MT004, MT028, and MT035), with 2.0 × 10^3^ ± 4.5 × 10^3^ CFU/g each. In the Negro River, the highest abundance was observed for *Colletotrichum* MT046 (320 ± 432 CFU/g). In the Tapajós River, *Penicillium* MT027 showed the highest value (800 ± 1304 CFU/g), followed by *Colletotrichum* MT046 (600 ± 1342 CFU/g).

In water (Table 6), mean abundance values were lower than those observed in solid substrates. The highest value in the Negro River was recorded for *Oidiodendron* MT067 (8 ± 8 CFU/mL), whereas in the Solimões and Tapajós Rivers, the highest abundances corresponded to *Rhizopus* MT015 (40 ± 89 CFU/mL) and yeast morphotype MT014 (42 ± 88 CFU/mL), respectively.

These results indicate that, in addition to differences in occurrence, morphotypes also exhibited distinct quantitative patterns depending on the respective substrate and river system, supporting likely influence of environmental conditions on fungal community structure.

### 3.5. Community Structure and Diversity

Community structure varied markedly among substrates and rivers. In submerged wood, the Negro River showed the highest Shannon diversity (H′ = 2.56) and high Pielou evenness (J = 0.83), together with one of the lowest Simpson dominance values (D = 0.10), whereas the Solimões River showed the lowest diversity (H′ = 1.23) and highest dominance (D = 0.47). The Tapajós River presented intermediate to high diversity (H′ = 2.43), low dominance (D = 0.11), and high evenness (J = 0.86).

In sediment, the Tapajós River exhibited the highest diversity (H′ = 2.63) and lowest dominance (D = 0.09), while the Solimões River again showed the lowest diversity (H′ = 1.93) and higher dominance (D = 0.19). The Negro River showed intermediate values of diversity (H′ = 2.41), low dominance (D = 0.12), and high evenness (J = 0.83).

In water samples, the Tapajós River also showed the highest diversity (H′ = 2.08), relatively low dominance (D = 0.16), and high evenness (J = 0.81), whereas the Negro River presented lower richness (S = 7) but high evenness (J = 0.91), indicating a more uniform distribution of morphotypes despite moderate diversity (H′ = 1.77). The Solimões River showed the lowest diversity (H′ = 1.68) and highest dominance (D = 0.22) among water samples.

Representative dominant morphotypes varied according to substrate and river system, with different morphotypes prevailing under each environmental condition. In submerged wood, the dominant morphotypes included *Acremonium* MT040, *Penicillium* MT027, and *Scedosporium* MT038 in the Solimões River; *Penicillium* MT028 and *Penicillium* MT033 in the Tapajós River, followed by co-dominant *Aspergillus* MT023 and *Paecilomyces* MT022; and *Aspergillus* MT001 and *Penicillium* MT003 in the Negro River, followed by co-dominant *Aspergillus* MT013 and *Nodulisporium* MT006. In sediment, dominant morphotypes were mainly *Penicillium* morphotypes in the Solimões River, while *Penicillium* MT027, *Penicillium* MT028, and *Colletotrichum* MT046 prevailed in the Tapajós River. In the Negro River sediment, *Colletotrichum* MT046 and sterile mycelium MT044 were followed by co-dominant *Aspergillus* MT001 and *Penicillium* MT005. In water, dominant morphotypes included *Rhizopus* MT015 and *Cladosporium* MT051 in the Solimões River, followed by co-dominant sterile mycelium MT066 and *Penicillium* MT057; yeast-like MT014 in the Tapajós River, followed by co-dominant *Mucor* MT075, *Penicillium* MT028, and *Rhizopus* MT015; and *Oidiodendron* MT067 and sterile mycelium MT042 in the Negro River, followed by several low-abundance morphotypes with similar mean values.

Similarity among rivers was low across all substrates, particularly in submerged wood, suggesting apparent compositional differentiation among river-associated cultivable fungal communities. In this substrate, the highest Sørensen similarity was observed between the Negro and Tapajós Rivers, whereas the lowest occurred between the Negro and Solimões Rivers. In sediment and water, the highest similarity values were recorded between the Solimões and Tapajós Rivers.

**Figure 3:**
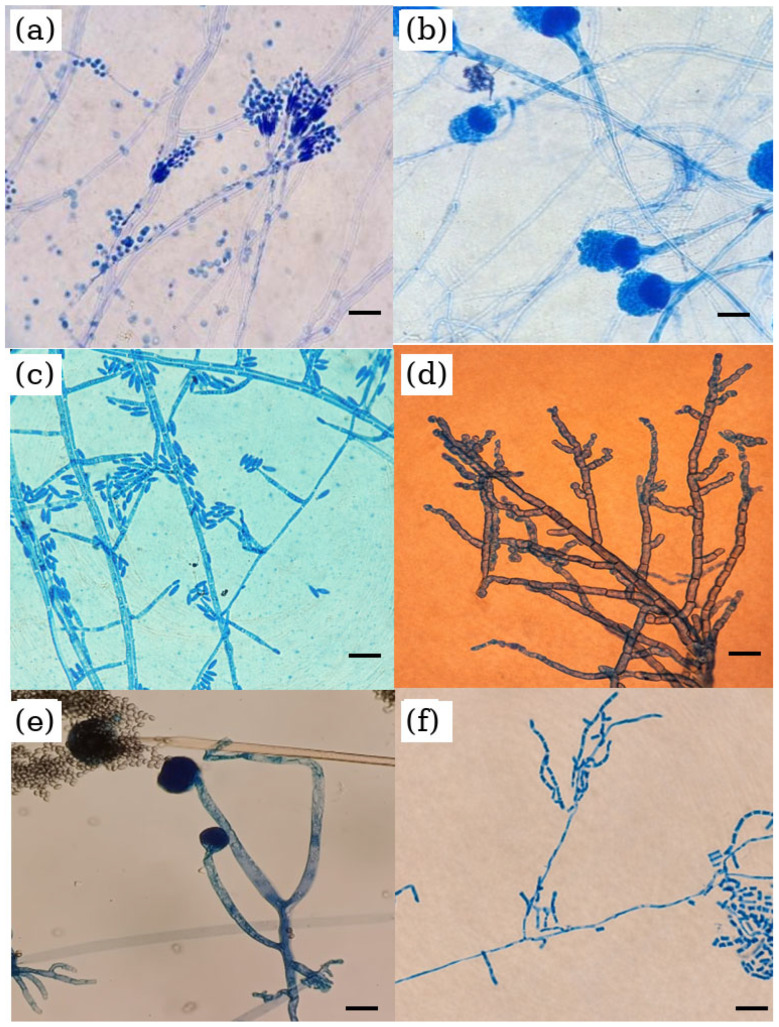
Representative micromorphological features used for genus of selected fungal isolates recovered from Amazonian river substrates. The panels show representative microscopic features of different fungal genera/morphotypes recovered in the study: (**a**) *Penicillium* sp.; (**b**) *Aspergillus* sp.; (**c**) *Acremonium* sp.; (**d**) *Colletotrichum* sp.; (**e**) *Rhizopus* sp.; and (**f**) *Oidiodendron* sp. Abbreviations: MT, morphotype; N, Negro River; S, Solimões River; T, Tapajós River. Scale bars = 10 µm.

Representative dominant morphotypes also differed across substrates and rivers (Figure 3). In wood, the highest mean abundances were recorded for *Acremonium* MT040 in the Solimões River, *Aspergillus* MT001 in the Negro River, and *Penicillium* MT028 in the Tapajós River. In sediment, *Penicillium* morphotypes predominated in the Solimões River, *Colletotrichum* MT046 was most frequent in the Negro River, and *Penicillium* MT027 was most abundant in the Tapajós River. In water, abundance values were generally lower, with *Oidiodendron* MT067, *Rhizopus* MT015, and yeast-like morphotype MT014 being the most abundant representative morphotypes in the Negro, Solimões, and Tapajós Rivers, respectively; additional co-dominant or low-abundance morphotypes are detailed in Table 7.

## 4. Discussion

In the present study, four main patterns were identified. First, cultivable fungi were more abundant and richer in solid substrates, particularly submerged wood and sediment, than in water samples. Second, the strongest abundance and richness patterns were substrate-dependent: submerged wood from the Negro River showed the highest overall fungal abundance and high diversity, whereas the Tapajós River showed the highest diversity and richness in water and sediment. Third, the Solimões River showed stronger dominance by a limited number of morphotypes, particularly in submerged wood, indicating a less even community structure under the applied culture conditions. Fourth, low Sørensen similarity values among rivers suggested apparent compositional differentiation, especially in submerged wood. A total of 130 isolates representing 75 morphotypes were recovered, with the highest abundance in submerged wood from the Negro River (3.4 × 10^4^ CFU/g) and the highest abundance in water from the Tapajós River (140 CFU/mL). These findings expand current knowledge of tropical freshwater fungi and support the combined influence of substrate type and hydrochemical gradients on cultivable fungal community structure.

Fungal richness was strongly influenced by substrate and river type. The Tapajós River showed the highest richness in water (13 morphotypes) and sediment (22 morphotypes), whereas the Negro River showed the highest richness in submerged wood (22 morphotypes). Because the river systems were sampled in different months, the sampling design should be interpreted as an exploratory cross-sectional comparison rather than as a temporally synchronized survey. Solid substrates consistently supported richness values similar to or higher than those of water. Similar trends were reported in Lake Wuchang and the Songhua River, where sediments harbored more diverse fungal communities than surface waters [29,30]. In Amazonian lakes, submerged wood also supported high richness of decomposer fungi [16]. These patterns suggest that nutrient retention, habitat stability, and microsite heterogeneity make solid substrates favorable environments for fungal colonization. Sediments may also function as reservoirs of spores and persistent propagules, as indicated by culture-based studies of sediment-associated mycobiota and by reports showing that sediment microbial communities respond strongly to organic matter gradients and watershed disturbance [31,32,33].

At the genus/morphotype level, the cultivable fraction was dominated by cosmopolitan filamentous genera, particularly *Aspergillus* and *Penicillium*, together with *Colletotrichum*, *Trichoderma*, *Rhizopus*, *Oidiodendron*, *Acremonium*, and sterile or yeast-like morphotypes. Submerged wood from the Negro River showed high abundances of *Aspergillus* MT001, whereas *Penicillium* MT028 dominated wood from the Tapajós River. Similar dominance of filamentous Ascomycota in woody freshwater substrates has been reported in the Amazon [16], Europe [10], and Asia [11]. These genera likely prevail because of rapid sporulation, broad physiological tolerance, and efficient colonization of organic substrates.

Diversity indices calculated from CFU-based abundance data suggested apparent structural differences among rivers and substrates. In submerged wood, the Negro River showed the highest Shannon diversity and evenness, whereas the Solimões River showed the highest dominance. In sediment and water, the Tapajós River showed the highest diversity values. Comparable responses have been reported in Chinese lakes and wetlands, where environmental conditions strongly shaped fungal diversity [27,28]. Water quality was also associated with fungal community structure in coastal watersheds [34]. In Amazonian rivers, high dissolved organic carbon in black-water systems [35] and the strong suspended sediment dynamics of white-water systems such as the Solimões [36] are additional environmental features likely to contribute to such contrasts. Hydraulic disturbance is also recognized as a driver of microbial shifts in river–lake systems [37]. The CFU-based approach used here provides quantitative information only for the cultivable fraction recovered under the incubation and medium conditions applied in this study; therefore, these values should not be interpreted as estimates of total fungal diversity, but rather as complementary data on viable, culturable propagules among substrates and river systems.

Low Sørensen similarity values suggested limited morphotype sharing among rivers. This was most evident in submerged wood, where the lowest similarity occurred between the Negro and Solimões Rivers. In sediment and water, the highest similarity values involved Solimões × Tapajós. Similar compositional patterns have been reported in European river–groundwater systems [38], Chinese freshwater ecosystems [29,30], and tropical elevational gradients from the Andes to the Amazon [3]. These findings suggest that Amazonian rivers may form ecological mosaics in which pH, dissolved organic matter, suspended solids, and hydrological dynamics promote local selection of fungal assemblages.

Several methodological limitations should be considered when interpreting the present results. First, culture-based methods recover only the cultivable fraction of fungal communities and may underestimate uncultivable, slow-growing, substrate-dependent, or cryptic taxa. Second, the three river systems were sampled at different months, with collections performed in April, May, and September 2025. Therefore, seasonal variation due to rainfall, hydrology, water level, temperature, and organic matter input may have influenced fungal community structure and cannot be fully separated from river type-associated effects. Third, sample transport and processing differed among rivers. Tapajós samples were refrigerated and processed approximately 24 h after collection, whereas Negro and Solimões samples were processed within less than 2 h. Although refrigeration was used to minimize biological changes, delayed processing may have influenced the recovery of viable propagules and should be considered when comparing abundance and richness among rivers. Fourth, the use of PDA supplemented with chloramphenicol may favor fast-growing filamentous fungi, including cosmopolitan genera such as *Aspergillus*, *Penicillium*, *Cladosporium*, and *Fusarium*. These genera are common in environmental substrates but may also occur as laboratory contaminants; therefore, their occurrence was interpreted conservatively as part of the cultivable morphotype assemblage recovered under the conditions used in this study. Finally, taxonomic assignments were based on macro- and micromorphological traits, without ITS sequencing for the full isolate set. Because phenotypic plasticity, morphological convergence, and cryptic diversity are common among fungi, the results should be interpreted primarily at the morphotype or genus level rather than as definitive species-level identifications. Future studies should combine synchronized seasonal sampling, standardized processing times, ITS sequencing of representative isolates, additional taxonomic markers when necessary, and high-throughput sequencing to validate taxonomic assignments and provide a broader estimate of fungal diversity in these Amazonian river systems.

Taken together, the results suggest that the Negro, Solimões, and Tapajós Rivers differ in the cultivable fungal morphotype assemblages recovered under the applied culture conditions, and that these patterns are most informative when substrate type is considered together with water type. However, because sampling months and processing conditions were not fully synchronized among the investigated rivers, these apparent river-associated patterns should be interpreted cautiously. Submerged wood and sediment consistently supported higher abundance and richness than water, while the observed patterns included high fungal abundance in Negro River wood, high diversity in Tapajós River water and sediment, stronger dominance in the Solimões River, and low compositional overlap among rivers. These results highlight Amazonian rivers as reservoirs of poorly explored cultivable fungal diversity with ecological and likely biotechnological relevance, while also emphasizing a need for synchronized seasonal sampling and molecular confirmation in future studies.

## 5. Conclusions

This study indicates that substrate type and water type are associated with the structure of cultivable fungal communities recovered from major Amazonian rivers. Submerged wood and sediment consistently supported higher fungal abundance and richness than water, confirming their importance as key substrates for the recovery of aquatic fungi. However, because the rivers were sampled at different months and processing times differed among sites, these patterns should be interpreted as exploratory and descriptive rather than as definitive river-specific effects. Thus, this study provides a baseline for the cultivable fraction of Amazonian freshwater fungi, while highlighting the need for future studies integrating culture-dependent methods, molecular identification of isolates, high-throughput sequencing, synchronized seasonal sampling, and standardized processing conditions to obtain a broader view of fungal diversity in these river systems.

Apparent river-associated patterns were also observed: the Negro River showed the highest overall fungal abundance in submerged wood, the Tapajós River showed the highest diversity in water and sediment, and the Solimões River showed stronger dominance due to a limited number of morphotypes. Low Sørensen similarity values among rivers further suggest that black-, white-, and clear-water systems may harbor distinct cultivable fungal morphotype assemblages, although temporal and logistical effects cannot be excluded. Future studies derived from this collection should prioritize ITS sequencing of representative isolates from each morphotype, complemented by additional markers when necessary, to validate genus-level assignments and resolve species-level diversity.

## Figures and Tables

**Table 1:** Physicochemical characteristics of water samples collected from the Negro, Solimões, and Tapajós Rivers.

Parameter	Negro River (Manaus, Amazonas)	Solimões River (Iranduba, Amazonas)	Tapajós River (Belterra, Pará)
Temperature (°C)	29.8 ± 1.5	30.3 ± 1.5	31.7 ± 1.6
pH	5.53 ± 0.28	6.65 ± 0.33	6.59 ± 0.33
Dissolved oxygen (mg/L)	5.50 ± 0.28	4.1–6.1 *	5.44 ± 0.27
Electrical conductivity (µS·cm^−1^)	9.30 ± 0.47	~80–100 *	13.72 ± 0.69
Turbidity (A600)	0.029 ± 0.001	0.032 ± 0.009	0.015 ± 0.019

Footnote: Data measured in situ are presented as mean ± standard deviation and were rounded according to the decimal resolution of the instrument used for each parameter. Values marked with an asterisk (*) were obtained from the literature [21,22] and are presented as reported by the original sources; therefore, they were not rounded according to the instrument used in the present study.

**Table 2:** Mean abundance of cultivable fungi recovered from water, sediment, and submerged wood in three Amazonian river systems.

River	Water (CFU/mL)	Sediment (CFU/g)	Submerged Wood (CFU/g)
Negro	22 ± 4.5	1.6 × 10^3^ ± 1.4 × 10^3^	3.4 × 10^4^ ± 0.9 × 10^4^
Solimões	124 ± 101	8.2 × 10^3^ ± 9.5 × 10^3^	1.0 × 10^4^ ± 1.5 × 10^4^
Tapajós	140 ± 177	4.8 × 10^3^ ± 1.5 × 10^3^	2.1 × 10^4^ ± 1.1 × 10^4^

Footnote: Values are presented as mean ± standard deviation (*n* = 5).

**Table 3:** Morphotype richness of cultivable fungi isolated from water, sediment, and submerged wood in the Negro, Solimões, and Tapajós Rivers.

Substrate	Negro	Solimões	Tapajós
Water	7	9	13
Sediment	18	11	22
Submerged wood	22	11	17

Footnote: Morphotype richness corresponds to the number of distinct morphotypes recorded for each substrate in each river.

**Table 4:** Mean abundance (±standard deviation) of cultivable fungal morphotypes isolated from submerged wood in the Negro, Solimões, and Tapajós Rivers, expressed as CFU/g (*n* = 5).

Morphotype	Negro (CFU/g)	Solimões (CFU/g)	Tapajós (CFU/g)
*Acremonium* MT040	–	6.8 × 10^3^ ± 15.2 × 10^3^	–
*Acremonium* MT041	–	200 ± 447	–
*Aspergillus* MT001	6.4 × 10^3^ ± 9.2 × 10^3^	–	–
*Aspergillus* MT013	4.0 × 10^3^ ± 8.9 × 10^3^	–	–
*Aspergillus* MT023	–	20 ± 45	2.4 × 10^3^ ± 4.3 × 10^3^
*Cladosporium* MT034	–	–	200 ± 447
*Fusarium* MT036	–	200 ± 447	–
Undetermined MT007	400 ± 894	–	–
Undetermined MT011	200 ± 447	–	–
Yeast MT014	2.0 × 10^3^ ± 4.5 × 10^3^	–	200 ± 447
Yeast MT019	2.0 × 10^3^ ± 4.5 × 10^3^	–	–
Sterile mycelium MT029	–	–	1.4 × 10^3^ ± 3.1 × 10^3^
Sterile mycelium MT039	–	400 ± 548	–
Sterile mycelium MT042	–	20 ± 45	–
*Mucor* MT037	–	200 ± 447	–
*Nodulisporium* MT006	4.0 × 10^3^ ± 5.5 × 10^3^	–	–
*Paecilomyces* MT022	200 ± 447	–	2.4 × 10^3^ ± 3.8 × 10^3^
*Penicillium* MT002	2.0 × 10^3^ ± 4.5 × 10^3^	–	1.0 × 10^3^ ± 2.2 × 10^3^
*Penicillium* MT003	4.2 × 10^3^ ± 5.3 × 10^3^	–	–
*Penicillium* MT004	2.2 × 10^3^ ± 4.4 × 10^3^	–	–
*Penicillium* MT005	600 ± 894	–	200 ± 447
*Penicillium* MT008	2.0 × 10^3^ ± 4.5 × 10^3^	–	–
*Penicillium* MT010	400 ± 894	400 ± 548	–
*Penicillium* MT012	400 ± 894	–	–
*Penicillium* MT016	200 ± 447	–	–
*Penicillium* MT017	200 ± 447	–	–
*Penicillium* MT018	200 ± 447	–	–
*Penicillium* MT024	–	–	400 ± 548
*Penicillium* MT027	–	1.4 × 10^3^ ± 3.1 × 10^3^	1.4 × 10^3^ ± 3.1 × 10^3^
*Penicillium* MT028	–	–	4.4 × 10^3^ ± 6.2 × 10^3^
*Penicillium* MT030	–	–	200 ± 447
*Penicillium* MT031	–	–	1.8 × 10^3^ ± 3.5 × 10^3^
*Penicillium* MT032	–	–	400 ± 894
*Penicillium* MT033	–	–	2.8 × 10^3^ ± 6.3 × 10^3^
*Penicillium* MT035	–	–	200 ± 447
*Phoma* MT043	–	20 ± 45	–
*Rhizopus* MT015	200 ± 447	–	–
*Scedosporium* MT038	–	600 ± 1342	–
*Trichoderma* MT009	400 ± 894	–	–
*Trichoderma* MT020	200 ± 447	–	–
*Trichoderma* MT021	2.0 × 10^3^ ± 4.5 × 10^3^	–	–
*Trichoderma* MT025	–	–	1.2 × 10^3^ ± 2.7 × 10^3^
*Trichoderma* MT026	–	–	600 ± 1342

Footnote: Values represent mean values ± standard deviation of morphotype abundance calculated from five subsamples per river. The symbol (–) indicates absence of the morphotype in the respective river. Repeated mean ± standard deviation values occur when different morphotypes show the same distribution pattern among the five subsamples after dilution correction. For example, values such as 200 ± 447 indicate detection in only one subsample and absence from the remaining subsamples.

**Table 5:** Mean abundance (±standard deviation) of cultivable fungal morphotypes isolated from sediment in the Negro, Solimões, and Tapajós Rivers, expressed as CFU/g (*n* = 5).

Morphotype	Negro (CFU/g)	Solimões (CFU/g)	Tapajós (CFU/g)
*Aspergillus* MT001	200 ± 447	–	–
*Aspergillus* MT013	–	–	40 ± 89
*Aspergillus* MT023	–	–	500 ± 485
*Aspergillus* MT058	–	–	100 ± 141
*Aspergillus* MT060	–	–	200 ± 447
*Aspergillus* MT062	–	–	200 ± 447
*Cladosporium* MT051	40 ± 55	–	–
*Colletotrichum* MT045	140 ± 207	–	–
*Colletotrichum* MT046	320 ± 432	–	600 ± 1342
*Colletotrichum* MT054	40 ± 89	–	–
*Exophiala* MT049	20 ± 45	–	–
*Fusarium* MT063	–	–	20 ± 45
Undetermined MT047	80 ± 84	–	–
Undetermined MT050	20 ± 45	–	–
Undetermined MT059	–	–	200 ± 447
Yeast MT056	–	–	120 ± 268
Sterile mycelium MT042	20 ± 45	200 ± 447	–
Sterile mycelium MT044	240 ± 434	200 ± 447	–
Sterile mycelium MT048	20 ± 45	–	–
Sterile mycelium MT066	–	400 ± 548	–
*Nodulisporium* MT006	–	–	200 ± 447
*Nodulisporium* MT053	100 ± 224	–	–
*Paecilomyces* MT061	–	–	20 ± 45
*Penicillium* MT004	20 ± 45	2.0 × 10^3^ ± 4.5 × 10^3^	–
*Penicillium* MT005	200 ± 447	–	280 ± 390
*Penicillium* MT010	–	–	200 ± 447
*Penicillium* MT017	40 ± 89	–	–
*Penicillium* MT024	–	200 ± 447	60 ± 134
*Penicillium* MT027	–	400 ± 548	800 ± 1304
*Penicillium* MT028	–	2.0 × 10^3^ ± 4.5 × 10^3^	680 ± 844
*Penicillium* MT031	–	200 ± 447	320 ± 460
*Penicillium* MT035	20 ± 45	2.0 × 10^3^ ± 4.5 × 10^3^	–
*Penicillium* MT055	20 ± 45	–	–
*Penicillium* MT057	–	–	40 ± 89
*Penicillium* MT064	–	–	200 ± 447
*Phoma* MT065	–	400 ± 894	–
*Rhizopus* MT015	–	–	20 ± 45
*Scopulariopsis* MT052	20 ± 45	–	–
*Trichoderma* MT009	–	–	40 ± 89
*Trichoderma* MT026	–	200 ± 447	20 ± 45

Footnote: Values represent mean values ± standard deviation of morphotype abundance calculated from five subsamples per river. The symbol (–) indicates absence of the morphotype in the respective river. Repeated mean ± standard deviation values occur when different morphotypes show the same distribution pattern among the five subsamples after dilution correction. For example, values such as 200 ± 447 indicate detection in only one subsample and absence from the remaining subsamples.

**Table 6:** Mean abundance (±standard deviation) of cultivable fungal morphotypes isolated from water in the Negro, Solimões, and Tapajós Rivers, expressed as CFU/mL (*n* = 5).

Morphotype	Negro (CFU/mL)	Solimões (CFU/mL)	Tapajós (CFU/mL)
*Acremonium* MT073	–	–	2 ± 4
*Aspergillus MT072*	–	–	4 ± 5
*Aspergillus* MT023	2 ± 4	2 ± 4	–
*Cladosporium* MT051	–	32 ± 66	2 ± 4
*Fusarium* MT074	–	2 ± 4	2 ± 4
Undetermined MT070	–	2 ± 4	4 ± 9
Undetermined MT071	–	–	8 ± 13
Yeast MT014	–	–	42 ± 88
Yeast MT069	2 ± 4	–	–
*Mucor* MT075	–	–	20 ± 45
Sterile mycelium MT042	4 ± 9	–	12 ± 11
Sterile mycelium MT066	–	20 ± 45	–
*Oidiodendron* MT067	8 ± 8	–	–
*Paecilomyces* MT022	–	–	2 ± 4
*Penicillium* MT004	–	–	2 ± 4
*Penicillium* MT028	–	4 ± 9	20 ± 45
*Penicillium* MT032	2 ± 4	–	–
*Penicillium* MT057	–	20 ± 45	–
*Phoma* MT068	2 ± 4	–	–
*Rhizopus* MT015	2 ± 4	40 ± 89	20 ± 45
*Trichoderma* MT009	–	2 ± 4	–

Footnote: Values represent mean values ± standard deviation of morphotype abundance calculated from five subsamples per river. The symbol (–) indicates absence of the morphotype in the respective river. Repeated mean ± standard deviation values occur when different morphotypes show the same distribution pattern among the five subsamples after dilution correction. For example, values such as 200 ± 447 indicate detection in only one subsample and absence from the remaining subsamples.

**Table 7:** Diversity indices, representative dominant morphotypes, and Sørensen similarity of cultivable fungi across substrates and Amazonian rivers.

Substrate	Parameter	Negro	Solimões	Tapajós
Water	Richness	7	9	13
	Shannon (H′)	1.77	1.68	2.08
	Simpson (D)	0.21	0.22	0.16
	Pielou (J)	0.91	0.76	0.81
	Representative dominant morphotypes	*Oidiodendron* MT067; sterile mycelium MT042; several low-abundance morphotypes	*Rhizopus* MT015; *Cladosporium* MT051; sterile mycelium MT066/*Penicillium* MT057	Yeast-like MT014; *Mucor* MT075/*Penicillium* MT028/*Rhizopus* MT015
	Sørensen similarity	0.118 (N × S); 0.190 (N × T); 0.455 (S × T)		
Sediment	Richness	18	11	22
	Shannon (H′)	2.41	1.93	2.63
	Simpson (D)	0.12	0.19	0.09
	Pielou (J)	0.83	0.80	0.85
	Representative dominant morphotypes	*Colletotrichum* MT046; sterile mycelium MT044; *Aspergillus* MT001/*Penicillium* MT005	*Penicillium* MT004; *Penicillium* MT028; *Penicillium* MT035	*Penicillium* MT027; *Penicillium* MT028; *Colletotrichum* MT046
	Sørensen similarity	0.276 (N × S); 0.100 (N × T); 0.303 (S × T)		
Submerged wood	Richness	22	11	17
	Shannon (H′)	2.56	1.23	2.43
	Simpson (D)	0.10	0.47	0.11
	Pielou (J)	0.83	0.51	0.86
	Representative dominant morphotypes	*Aspergillus* MT001; *Penicillium* MT003; *Aspergillus* MT013/*Nodulisporium* MT006	*Acremonium* MT040; *Penicillium* MT027; *Scedosporium* MT038	*Penicillium* MT028; *Penicillium* MT033; *Aspergillus* MT023/*Paecilomyces* MT022
	Sørensen similarity	0.061 (N × S); 0.205 (N × T); 0.143 (S × T)		

Footnote: Richness, Shannon diversity, Simpson dominance, Pielou evenness, and Sørensen similarity values were calculated from abundance or presence/absence matrices. Representative dominant morphotypes were selected based on the highest mean abundance values; tied or near-tied morphotypes are discussed in the text when relevant. H′ = Shannon diversity index; D = Simpson dominance index; J = Pielou evenness index. Sørensen similarity (Cs) was calculated from presence/absence data. N = Negro River; S = Solimões River; T = Tapajós River.

## Data Availability

The datasets generated and analyzed during the current study are available in the Supplementary Materials associated with this article.

## Supplementary Materials

The following supporting information can be downloaded at: https://www.mdpi.com/article/10.3390/biology15120931/s1. Figure S1: Representative photographic records of field activities conducted in the Amazonian Tapajós, Negro, and Solimões rivers. Figure S2: Initial isolation plates of cultivable fungi obtained from environmental samples collected from the Negro, Tapajós, and Solimões rivers. Figure S3: Macromorphological appearance of the most abundant fungal morphotypes isolated from Amazonian environmental samples. Figure S4: Micromorphological characteristics of the most frequent filamentous fungi isolated from environmental samples of the Amazonian rivers Negro, Tapajós and Solimões, observed by microculture and stained with lactophenol cotton blue.

## Abbreviations

The following abbreviations are used in this manuscript:

AM	Amazonas
a.u.	Absorbance units
CFU	Colony-forming units
CNPq	National Council for Scientific and Technological Development
Cs	Sørensen similarity coefficient
D	Simpson dominance index
H’	Shannon diversity index
INPA	National Institute of Amazonian Research
J	Pielou evenness index
MCTI	Ministry of Science, Technology and Innovation
NaCl	Sodium chloride
PA	Pará
PDA	Potato dextrose agar
SisGen	National System of the Management of Genetic Heritage and Associated Traditional Knowledge
UEA	Amazonas State University
UFOPA	Federal University of Western Pará
