# Supplementary material for: Cultivable Fungi in Amazonian Black-, White-, and Clear-Water Rivers
Source: Biology (Basel). 2026 Jun 15;15(12):931. doi: 10.3390/biology15120931 (PMC13295409; doi:10.3390/biology15120931)
Supplement: Supplementary file 1 [file biology-15-00931-s001.zip › biology-4336089-supplementary.pdf]

Supplementary Material S1 - Additional photographic records of field sampling, fungal isolation, and morphological characterization of cultivable fungi from Amazonian river substrates.

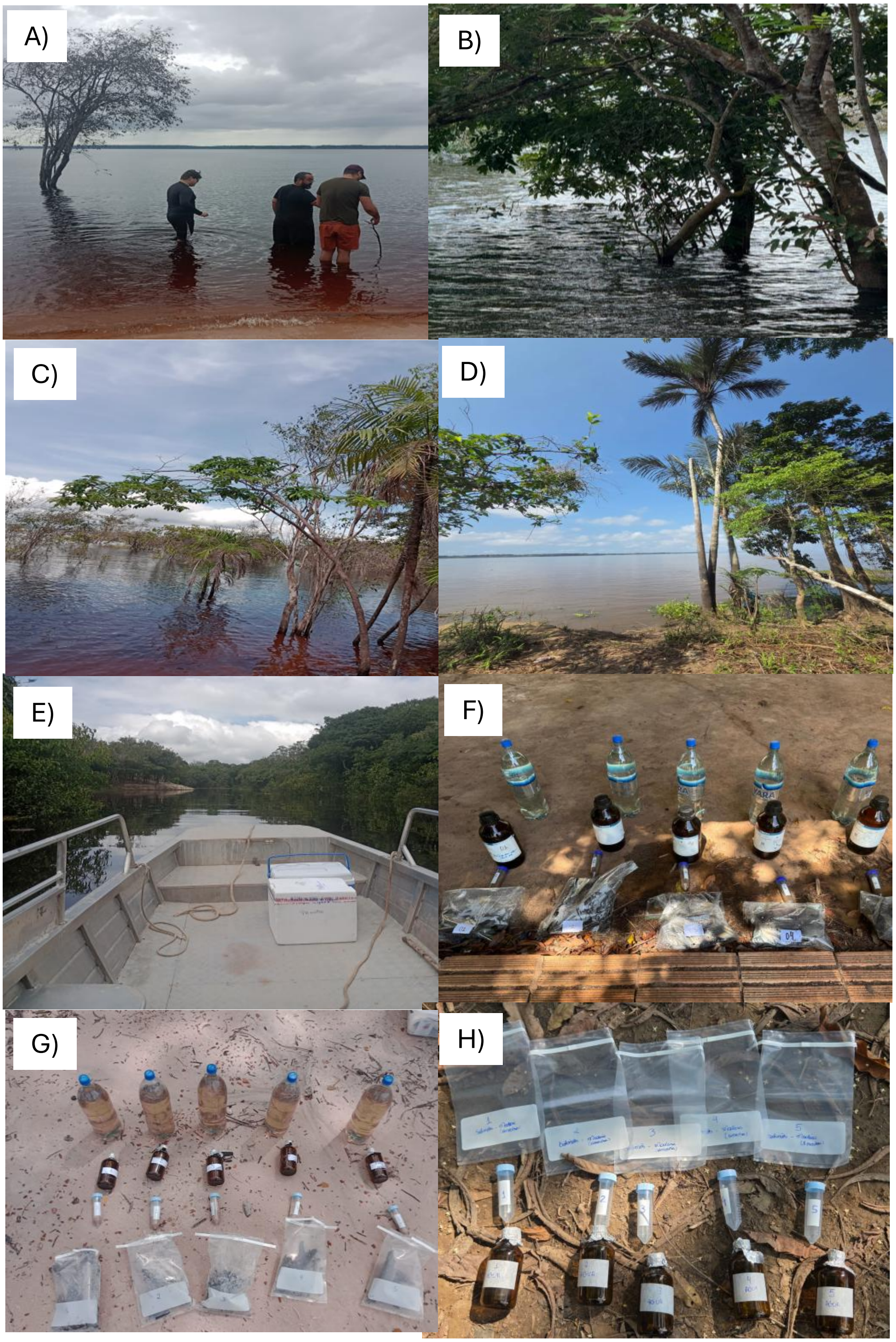

Figure S1. Representative photographic records of field activities conducted in the Amazonian Tapajós, Negro, and Solimões rivers.
(A) Team collecting environmental samples; (B) sampling site on the Tapajós River; (C) sampling site on the Negro River; (D) sampling site on the Solimões River; (E) boat used to access sampling sites in Amazonian floodplain forest environments; (F) environmental samples collected from the Tapajós River; (G) environmental samples collected from the Negro River; (H) environmental samples collected from the Solimões River.

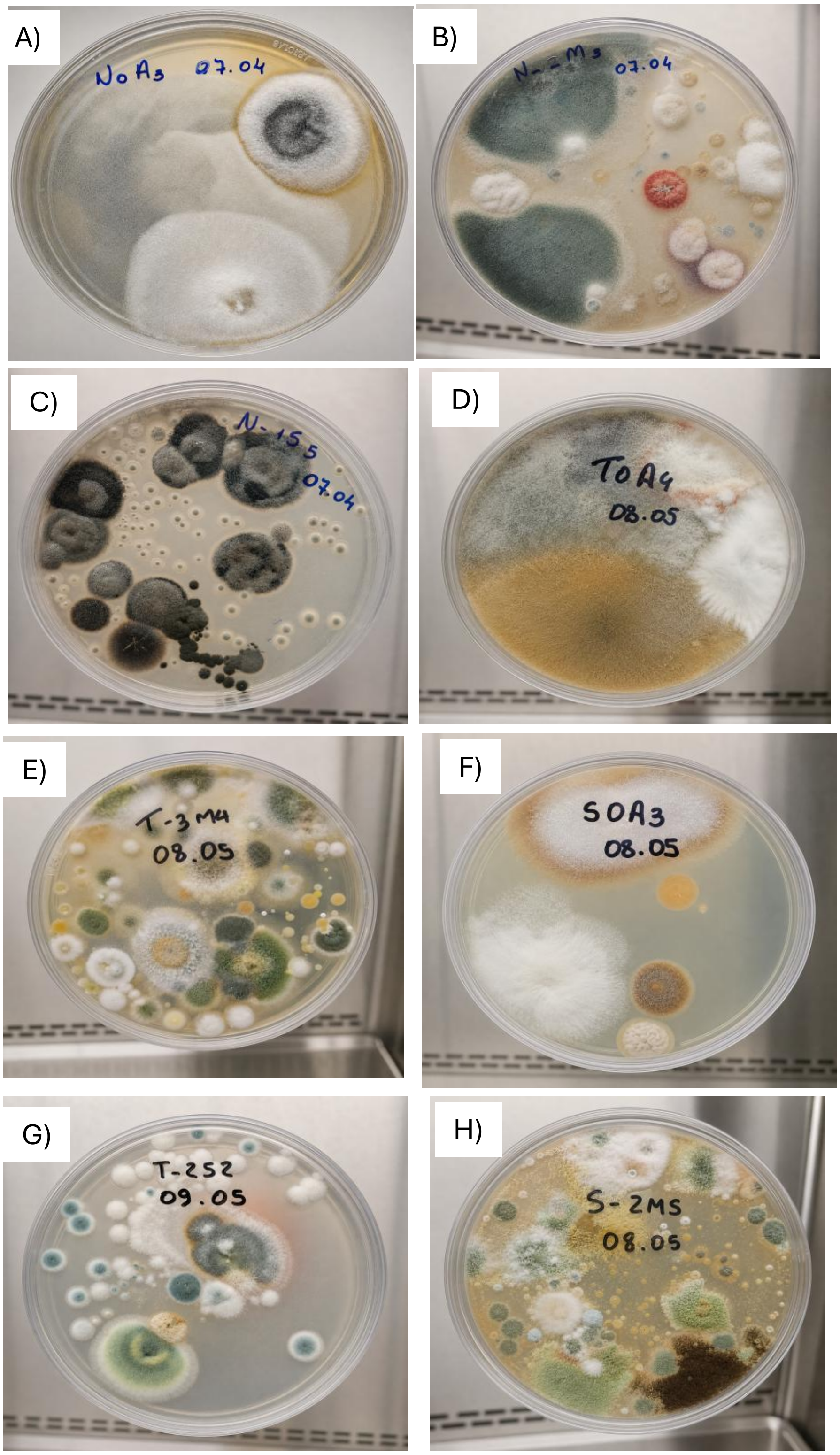

Figure S2. Initial isolation plates of cultivable fungi obtained from environmental samples collected from the Negro, Tapajós, and Solimões rivers.
The plates show fungal growth from water, sediment, and submerged wood samples.
(A) Negro River: water sample;
(B) Negro River: submerged wood sample;
(C) Negro River: sediment sample;
(D) Tapajós River: water sample;
(E) Tapajós River: sediment sample;
(F) Tapajós River: submerged wood sample;
(G) Solimões River: water sample;
(H) Solimões River: sediment sample.

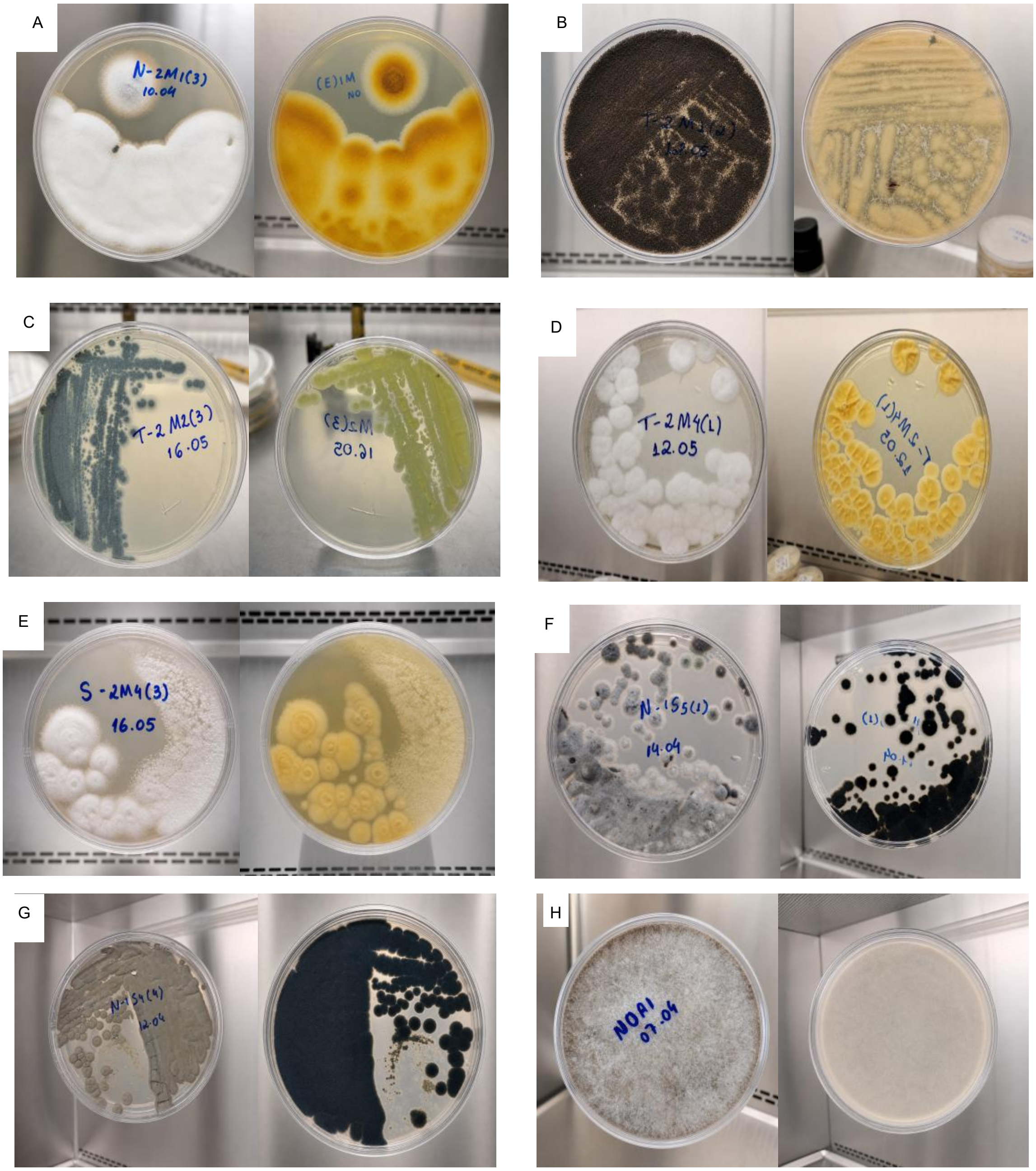

Figure S3. Macromorphological appearance of the most abundant fungal morphotypes isolated from Amazonian environmental samples.
Each panel shows the upper surface and reverse side of colonies grown on Potato Dextrose Agar (PDA).
(A) *Aspergillus* MT001;
(B) *Aspergillus* MT023;
(C) *Penicillium* MT027;
(D) *Penicillium* MT028;
(E) *Acremonium* MT040;
(F) *Colletotrichum* MT046;
(G) *Cladosporium* MT051;
(H) *Rhizopus* MT015.

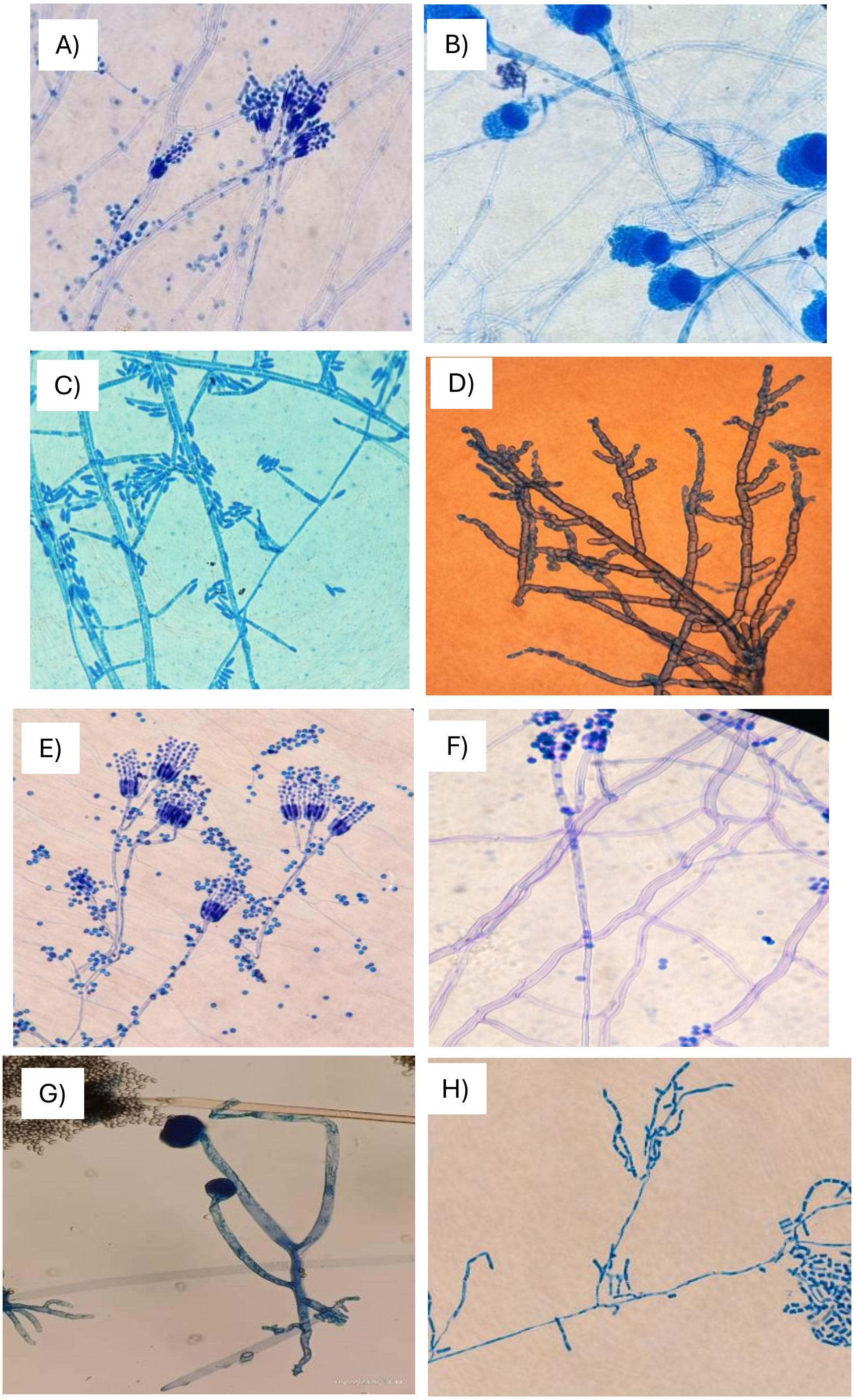

Figure S4. Macromorphological appearance of the most abundant fungal morphotypes isolated from Amazonian environmental samples.
Each panel shows the upper surface and reverse side of colonies grown on Potato Dextrose Agar (PDA).
(A) *Aspergillus* MT001;
(B) *Aspergillus* MT023;
(C) *Penicillium* MT027;
(D) *Penicillium* MT028;
(E) *Acremonium* MT040;
(F) *Colletotrichum* MT046;
(G) *Cladosporium* MT051;
(H) *Rhizopus* MT015.
